# Virtual Reality Intervention for Managing Apathy in People With Cognitive Impairment: Systematic Review

**DOI:** 10.2196/35224

**Published:** 2022-05-11

**Authors:** Ka Ying Ho, Po Mang Cheung, Tap Wing Cheng, Wing Yin Suen, Hiu Ying Ho, Daphne Sze Ki Cheung

**Affiliations:** 1 School of Nursing The Hong Kong Polytechinc University Hong Kong Hong Kong

**Keywords:** virtual reality, apathy, cognitive impairment, dementia, systematic review

## Abstract

**Background:**

Apathy is common in people with cognitive impairment. It leads to different consequences, such as more severe cognitive deficits, rapid functional decline, and decreased quality of life. Virtual reality (VR) interventions are increasingly being used to manage apathy in individuals with cognitive impairment. However, reports of VR interventions are scattered across studies, which has hindered the development and use of the interventions.

**Objective:**

This study aimed to systematically review existing evidence on the use of VR interventions for managing apathy in people with cognitive impairment with regard to the effectiveness, contents, and implementation of the interventions.

**Methods:**

The PRISMA (Preferred Reporting Items for Systematic Reviews and Meta-Analyses) guidelines were followed. The PubMed, Embase, CINAHL, and PsycINFO databases were systematically searched for experimental studies published up to March 13, 2022, that reported the effects of VR interventions on apathy in older adults with cognitive impairment. Hand searching and citation chasing were conducted. The results of the included studies were synthesized by using a narrative synthesis. Their quality was appraised by using the Effective Public Health Practice Project quality assessment tool. However, because the VR interventions varied in duration, content, and implementation across studies, a meta-analysis was not conducted.

**Results:**

A total of 22 studies were identified from the databases, of which 6 (27%) met the inclusion criteria. Of these 6 studies, 2 (33%) were randomized controlled trials, 1 (17%) was a controlled clinical trial, and 3 (50%) were quasi-experimental studies. Individual studies showed significant improvement in apathy and yielded within-group medium to large effect sizes. The level of immersion ranged from low to high. Minor adverse effects were reported. The VR content mostly included natural scenes, followed by city views and game-based activities. A background soundtrack was often used with natural scenes. Most (5/6, 83%) of the studies were conducted in a residential care setting and were implemented by health care professionals or researchers. Safety precautions were taken in most (5/6, 83%) of the studies.

**Conclusions:**

Although preliminary evidence shows that VR interventions may be effective and feasible for alleviating apathy in people with cognitive impairment, the methodological limitations in the included studies make it difficult to reach a firm conclusion on these points. The implementation of the interventions was highlighted and discussed. More rigorous studies are encouraged.

**Trial Registration:**

PROSPERO International Prospective Register of Systematic Reviews CRD42021268289; https://www.crd.york.ac.uk/prospero/display_record.php?ID=CRD42021268289

## Introduction

### Background

Apathy is defined as an observable behavioral syndrome that is reflected in a reduction in goal-directed behaviors (as indicated by a lack of effort, initiative, perseverance, and productivity) [1]. Apathy is found in 2% to 75.2% of patients with cognitive impairments [2,3]. It is associated with suspected lesions in the prefrontal cortex and basal ganglia, which reduces a patient’s ability to initiate, sequence, and complete tasks, thereby affecting their everyday activities and autonomy [4-7]. As a result, patients who have developed apathy have exhibited more severe cognitive deficits, rapid functional decline, and decreased quality of life than the general population [3,8,9].

Virtual reality (VR) interventions are increasingly being used in caring for people with cognitive impairment. VR can be defined as a computer technology that reproduces a real or imaginary environment and simulates the user’s presence in that physical environment; therefore, the user would have a feeling of *being there* and be able to interact with the virtual environment through the engagement of their senses [10,11]. The level of immersion can be classified as low, moderate, or high [12]. Using a head-mounted display (HMD) or surround projection can be classified as being of a high level of immersion, defined as including more than 2 sensory modalities (eg, visual, auditory, and proprioceptive or motor) and stimuli that are oriented spatially. A moderate VR immersion level accommodates 1 to 2 sensory modalities with large-screen projection and stimuli, which may or may not be oriented spatially. A low VR immersion level only accommodates 1 sensory modality. A higher level of immersion is suggested because the patient may feel a higher level of presence, thus substantially increasing the behavioral responses [13,14].

How VR interventions alleviate apathy can be explained by a biomedical model and a psychosocial model. The biomedical model suggests that VR allows users to interact with a virtual, enriched environment, which triggers the reorganization and reconstruction of new cellular synapses to repair the brain lesions causing apathy, which refers to the neuroplasticity of the brain and nervous system [15]. When users receive multisensory feedback, they experience the illusion of place and then respond to the virtual environment as they would to the real world, resulting in better performance with more intensive, repetitive, and engaging experiences [15,16]. The psychosocial model suggests that users perceive themselves as being present in the virtual world [17,18] and that this immersion is envisaged as a mental sensation of engagement that would promote motivation [17]. VR achieves immersion by removing real-world sensations that individuals might not be able to process because of cognitive impairment and replacing them with virtual experiences. This gives a specific kind of stimulation, making it easier for users to focus and forget about their actual surroundings, thereby facilitating involvement.

VR may be an effective, customizable, and affordable solution for managing apathy in patients with cognitive impairment. For example, VR increases a sense of reality through digital media over tangible prompts in reminiscence therapy, thus increasing therapy effectiveness and treatment compliance [19,20]. As an accessible, low-cost, and customizable solution, VR also provides an alternative to *live music therapy*, which has been proven to be an effective solution for managing apathy but is too expensive and complicated to organize when a social distancing policy is in place [21-23]. Hence, in recent years, there have been an increasing number of studies investigating the benefits of VR interventions for patients with cognitive impairment. However, the reports on the apathy outcomes of VR interventions are scattered across studies. There is inconsistent evidence regarding the effectiveness of VR interventions [24-29], making the evaluation of effects difficult. Their study designs, VR contents, and implementation procedures have also differed; in addition, they have not been systematically reviewed in terms of quality. This has hindered the development of VR interventions and their adoption in practice.

### Objectives

This systematic review aimed to address this knowledge gap by reviewing the existing evidence on the use of VR interventions for managing apathy in people with cognitive impairment. The objectives of this review are to (1) evaluate the effects of VR interventions for managing apathy in people with cognitive impairment, (2) identify the content of the VR interventions for managing apathy in people with cognitive impairment, and (3) understand the implementation of VR interventions for managing apathy in people with cognitive impairment.

## Methods

### Design

This study was conducted with reference to the PRISMA (Preferred Reporting Items for Systematic Reviews and Meta-Analyses) statement [30,31]. The review protocol has been registered with PROSPERO (CRD 42021268289).

### Eligibility Criteria

The eligibility criteria are listed herein. The inclusion criteria were set according to the Population, Intervention, Comparison, Outcome, and Study design framework [32]. Studies that met the following criteria were included: (1) population: older adults with cognitive impairment, including those with subjective cognitive decline, mild cognitive impairment, and dementia; (2) intervention: a VR intervention using either immersive-type or on-screen approaches; (3) comparison: an active, a passive, or no control group for comparison; (4) outcome: apathy measured quantitatively with validated instruments; (5) study design: a randomized controlled trial or quasi-experimental study; and (6) published in English.

The exclusion criteria were as follows: (1) conference abstracts and reviews or (2) any article involving a multi-domain intervention with other modalities, such as augmented reality, because in such a situation, it would not be possible to attribute the reported intervention effect solely to the VR intervention.

### Sources of Information

In total, 4 databases (PubMed, Embase, CINAHL, and PsycINFO) were searched from inception to March 13, 2022. These databases are relevant to the research questions because PubMed focuses on biomedicine and health, Embase contributes to the biomedical research community by providing information and showing biomedical evidence to support essential life sciences functions, CINAHL contains articles on a wide range of topics such as nursing and biomedicine, and PsycINFO mainly covers journal articles in psychology and related disciplines.

To minimize the possibility that relevant articles not published in these 4 databases might be overlooked, the reference lists of the included studies were screened against the same set of eligibility criteria and included if relevant. Hand searching of articles in Google Scholar was also performed.

### Search Strategies

The following search strategy was developed with reference to the research questions and refined with the support of a university librarian (Pao Yue-Kong Library, Hong Kong Polytechnic University). The search keywords are listed herein (Multimedia Appendix 1 provides details of the search conducted in each database):

1. [Dementia]
  OR
  [Cognitive impairment]
  OR
  [Alzheimer disease]
  OR
  [Mild cognitive impairment]
  AND
2. [Virtual Reality]
  OR
  [Head mounted]
  OR
  [Simulation]
  OR
  [Virtual]
  AND
3. [Apathy]
  OR
  [Apathetic]
  OR
  [Lack of initiati*]
  OR
  [Lack of interest]

### Selection Process

Articles retrieved from the databases were managed using EndNote (version 20.0; Clarivate). Duplicate articles were first removed. Next, the screening of titles and abstracts against the eligibility criteria was conducted independently by the first and second authors (KYH and PMC, respectively). Subsequently, full-text screening was carried out independently by the third and fourth authors (TWC and WYS, respectively). Any disagreements over the reviews were resolved by consensus and by discussion with the fifth author (HYH).

### Data Extraction Process and Data Items

Each study was evaluated independently and duplicated using a pretested standardized data extraction form. For each study, the following information was extracted: (1) publication data (ie, year, author, and title), (2) study design, (3) setting, (4) sample, (5) VR intervention components, (6) outcomes, and (7) adverse events.

### Risk of Bias and Quality Assessment

The Effective Public Health Practice Project quality assessment tool was used to assess the risk of bias in the included studies [33]. In total, 6 domains of bias were scored with three rating categories: (1) strong, (2) moderate, and (3) weak. The 6 domains of bias are selection bias, study design, confounders, blinding, data collection method, and withdrawals and dropouts. The global rating of the study is strong if there is no weak rating in all components, whereas the study quality is rated as moderate or weak if there is 1 weak rating or ≥2 weak ratings, respectively. All the selected articles were scored by 2 authors in duplicate and independently. Disagreements were resolved through discussion.

### Effect Measures

Apathy is the outcome of interest that can be assessed quantitatively using validated instruments; for example, the Neuropsychiatric Inventory (Apathy subscale), the Apathy Evaluation Scale, the Structured Clinical Interview for Apathy, and the Dementia Apathy Interview and Rating Scale [34]. Continuous outcomes of apathy were reported by means and SDs. The corresponding within-group effect size of the intervention on apathy reported in the individual studies was calculated.

### Synthesis

The data extracted from the individual studies were narratively synthesized. These descriptions facilitated the examination of patterns across studies in a systematic manner. A meta-analysis was not conducted because of the heterogeneity of the included studies [23].

## Results

### Study Selection

**Figure 1:**
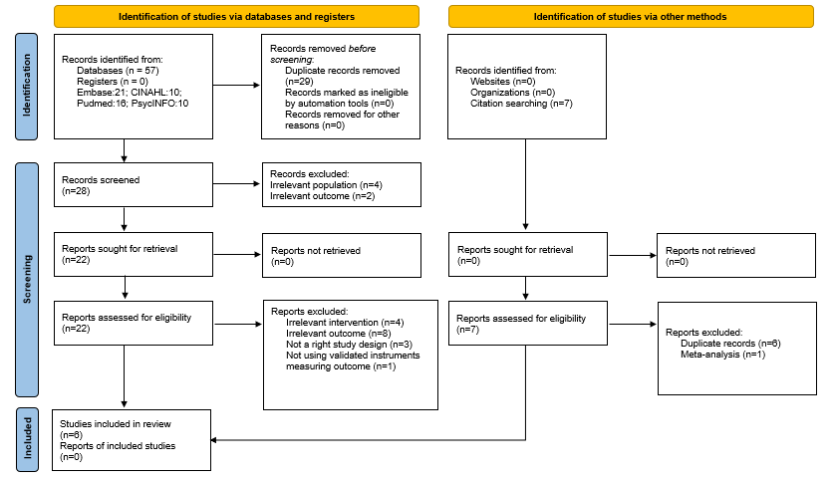
PRISMA (Preferred Reporting Items for Systematic Reviews and Meta-Analyses) flowchart.

As shown in Figure 1 [35], a total of 57 articles were identified from the databases; after removing duplicates, the titles and abstracts of 28 (49%) were screened. Of these 28 articles, 6 (21%) were excluded (n=4, 67%, because of incorrect study population and n=2, 33%, because of irrelevant study outcome), leaving the full text of 22 (79%) to be screened against the eligibility criteria. Of these 22 articles, 16 (73%) were excluded because they involved an irrelevant intervention (n=4, 25%), had an irrelevant outcome (n=8; 50%), did not use validated instruments measuring the outcome (n=1, 6%), or did not have the right study design (n=3, 19%). After searching the citations in the included articles, no further eligible articles were identified. In the end, of the 57 articles identified from the databases, 6 (11%) were included in this review.

### Study Characteristics

The characteristics of the included studies are presented in Table 1. Among the 6 included studies, 2 (33%) were randomized controlled trials (parallel-group and crossover design each) [26,27], 1 (17%) was a nonequivalent group controlled trial [29], and the remaining 3 (50%) were quasi-experimental studies with a single-group pre- and posttest design [24,25,28]. Most (5/6, 83%) of the studies were conducted in Australia [24-26,28,29], and 1 (17%) was conducted in South Korea [27]. Of the 6 studies, almost all (n=5, 83%) were conducted in a residential care setting [24-26,28,29], except for 1 (17%) that was conducted at a memory clinic [27]. The number of participants ranged from 10 to 46. All participants had various degrees of cognitive impairment, ranging from subjective cognitive decline to severe dementia. Only 17% (1/6) of the studies clearly defined apathy as “a lack of interest and diminished motivation” [29]. Of the 6 studies, 4 (67%) used the Person-Environment Apathy Rating Scale [24-26,28] to measure the apathy level before and during the VR intervention and 2 (33%) adopted the Apathy Evaluation Scale to measure apathy before and after the VR intervention [27,29].

**Table 1:** Characteristics of the included studies (N=6).

Study, country	Study design	Setting; sample	VR^a^ content; facilitator background	Device or devices; immersion level	Dosage	Outcome measurement and main findings	Adverse events
Brimelow et al, 2020 [24], Australia	QERMSG^b^	RACF^c^; mild to severe cognitive impairment (n=13)	360^o^ video relaxing scenes, leisure and lifestyle coordinator	Samsung Galaxy S7 and Samsung Gear VR headset; high	One 4-5 minutes	PEAR^d^; mean total score: before: 15.54 (SD 6.11), during: 11.38 (SD 3.93)*; P*=.005; within group effect size *r*=0.78	Blurring vision; headset-related discomfort
Brimelow et al, 2021 [25], Australia	QERMSG	RACF Mild to severe cognitive impairment (n=25)	Participant preferred natural scenery and household; OT^e^ and RN^f^	Samsung Galaxy S7 and Samsung Gear VR headset; high	Six 10-minutes over 3 weeks	PEAR; mean Apathy Subscale score: before and during: no information given; *P*<.001; within group effect size *r*=0.56	Mild headache; giddiness sensation; headset-related discomfort
D’Cunha et al, 2020 [26], Australia	Mixed methods crossover RCT^g^	RACF; mild to severe cognitive impairment (n=11)	Virtual cycling to simulate paddling in a lake or biking in a mountain; OT	Projector screen and pedal exercisers (Body Charger GB3030 UBE); moderate	One 25-mins	PEAR; mean Apathy Subscale score: control: 13.4 (SD 2.72), during VR: 12.6 (SD 2.37)*; P*=.49; between-group effect size *g*=0.31	Lower body discomfort during cycling
Kang et al, 2021 [27], South Korea	RCT	Memory clinic; SCD^h^ and MCI^i^ (n=45)	Multiple cognitive games; clinical neuropsychologist	Head-mounted Oculus Rift CV1 display; high	Eight 20-30 minutes	AES^j^; mean score: VR group, before: 47.43 (SD 10.20), after: 54.35 (SD 9.41), within group comparison *P*=.006; within group effect size *g*=0.68; control group, before: 52.83 (SD 9.38), after: 51.22 (SD 8.72), group×time effect size n^2^=0.17; group×time interaction *P*=.01	Nausea, oculomotor discomfort, and disorientation
Moyle et al, 2018 [28], Australia	QERMSG	RACF; dementia (n=10)	VR forest; trained care worker	Large interaction-enabled screen display and kinectmotion sensors; moderate	One 15 minutes	PEAR; mean Apathy Subscale score: before: 18.30 (SD 5.10); during: 12.10 (SD 2.69); after: 18.70 (SD 4.24); before-during: *P*=.01, effect size *g=*1.39; before-after: *P*>.05, effect size *g*=0.08	Not reported
Saredakis et al, 2021 [29], Australia	Nonequivalent group controlled trial	RACF; Minimal to moderate cognitive impairment (n=46)	Wander (Parkline Interactive), YouTube VR (Google LLC); researcher	VR group: Oculus Go HMD^k^ and laptop group: laptop computer; low	Three 20-minutes	AES mean score: VR group, before: 35.3 (SD 8.7), after: 36.0 (SD 6.1); within group effect size *g*=−0.09; laptop group: before: 41.8 (SD 7.1), after: 40.2 (SD 8.1), within group effect size *g*=0.20; control group, before: 44.3 (SD 9.5), after: 43.6 (SD 9.4); within group effect size *g*=0.1; time×(VR and laptop groups vs control group); *P*=.88; effect size n^2^=.00; time×(VR vs laptop group); *P*=.24; effect size n^2^=.03	Headache, heavy head feeling

^a^VR: virtual reality.

^b^QERMSG: quasi-experimental repeated measures single-group.

^c^RACF: residential aged care facilities.

^d^PEAR: Person-Environment Apathy Rating Scale.

^e^OT: occupational therapist.

^f^RN: registered nurse.

^g^RCT: randomized controlled trial.

^h^SCD: subjective cognitive decline.

^i^MCI: mild cognitive impairment.

^j^AES: Apathy Evaluation Scale.

^k^HMD: head-mounted display.

### Effects of the VR Interventions

Of the 6 included studies, 4 (67%) reported a significant positive within-group improvement in apathy during [24,25,28] or after [27] the VR intervention and yielded a medium to large effect size (0.56 to 1.39), whereas 2 (33%) reported no significant improvement [26,29].

Whereas 67% (4/6) of the studies used HMDs to implement a high VR immersion level [24,25,27,29], 33% (2/6) used a large-screen display to implement a moderate VR immersion level [26,28] and 17% (1/6) used a laptop computer to implement a low VR immersion level [29]. Large-screen displays along with pedal exercisers were included in 17% (1/6) of the studies [26].

Of the 6 included studies, only 1 (17%) compared the effects of a VR intervention delivered with a high-immersion HMD with those of a VR intervention delivered with a low-immersion laptop computer, but it did not show a significant difference between the 2 groups. Instead, the 2 VR groups still showed a significantly better improvement than the passive control group [29].

Mild adverse reactions were reported in 67% (4/6) of the studies, including eyestrain, blurred vision, and discomfort induced by a weighty headset [24,25,27,29].

### Content of the VR Interventions

In 67% (4/6) of the studies, there were natural scenes in the VR content, including underwater themes, beaches, farmyard animals, travel destinations, snowscapes, lakes, and mountain views [24-26,28], whereas 33% (2/6) of the studies included games and customized images related to personal experience (eg, home and school) [27,29]. Animals and natural scenes dominated the participants’ choices. Some participants reported that they found the realistic, colorful scenery visually appealing and had developed a sense of being outdoors [24-26,28]; a few participants preferred content that allowed interaction with the system (eg, challenges or tasks), whereas others did not [28]. Most of the participants preferred having customized content and a list of scene choices so that they could select content based on their own interests [24,25,28,29]. Apart from the natural scenes, 50% (3/6) of the studies combined the VR scenes with some background soundtrack or narration; for example, a forest scene with bird calls and travel destinations with music that participants had memories of [24,28,29]. Of the 6 included studies, 1 (17%) combined VR with a cycling experience, which allowed participants to paddle around a familiar lake and travel on a downhill path on a mountain biking track [26], and 1 (17%) consisted of multiple games involving 8 multi-domain cognitive tasks that allowed participants to exercise their visuospatial skills through learning and transference outcomes [27].

Of the included studies, 50% (3/6) delivered the VR intervention once, with the duration ranging from 4 to 25 minutes [24,26,28], whereas 50% (3/6) reported that there were 3 to 8 sessions and that the duration of each session ranged from 10 to 30 minutes [25,27,29].

### Implementation of the VR Intervention

In 67% (4/6) of the studies, the VR intervention was delivered by health care professionals such as registered nurses, occupational therapists, clinical neuropsychologist, and researchers [24,26,27,29], whereas in 33% (2/6), the VR intervention was implemented by frontline care workers working at residential aged care facilities [25,28].

In the studies (4/6, 67%) using high-immersion VR, the following hardware equipment was used: a Samsung Gear VR headset, an Oculus Quest HMD, and an Oculus Rift CV1 HMD with Oculus Touch controllers held by the participants with both hands [24,25,27,29]. Videos were presented on projector screens in 33% (2/6) [26,28] of the studies and on a laptop computer in 17% (1/6) [29] of the studies, which were of moderate and low immersion levels, respectively.

Safety was the top priority in the implementation of the VR interventions. Researchers included different safety measures in their studies. For instance, older adults with vision impairment and incomplete range of motion in their hips, knees, and ankles were excluded at recruitment [25,26,28]. A safety protocol was developed for use by the care staff and an on-site physiotherapist [26]. Furthermore, the videos used in the VR intervention did not contain sudden scene changes to reduce the risk of cybersickness [24,25,27,29]. In 50% (3/6) of the studies, the participants were instructed to remain seated throughout the whole experience to reduce the risk of falls [24,27,29].

### Study Quality

The quality ratings of the included studies are presented in Table 2. Of the 6 included studies, 5 (83%) were rated as *weak* and 1 (17%) was rated as *moderate* according to the Effective Public Health Practice Project quality assessment tool. The quality was generally low, mainly because of the unrepresentativeness of the target population, uncontrolled confounders, or a lack of blinding.

In all the included studies, participants were recruited from 1 to 3 residential aged care facilities or clinics by convenience sampling. A formal power analysis to estimate the sample size was not performed in any of the included studies. Of the 6 studies, 2 (33%) included >40 participants [27,29], whereas the other 4 (67%) had small samples [24-26,28]. Of the 6 studies, only 2 (33%) identified their confounders [26,27]; none reported adjusting for confounders such as gender, age, education level, and health status, which potentially threatened the validity of the results. Concerning blinding, there was no mention in any of the studies of whether the outcome assessors or participants were blinded to the group allocation and the research question. This is likely because blinding the participants is impossible because of the nature of VR interventions. Nevertheless, the included studies measured apathy with validated instruments. Therefore, we were convinced of the appropriateness of the data collection methods.

Some common risks of bias across different studies have been identified. Some items might not have been included in the research findings because of selective reporting. For instance, missing or insufficient data and some negative research findings might have been excluded by the authors. Publication bias is also possible.

**Table 2:** Assessment of the quality of the included studies using the Effective Public Health Practice Project quality assessment tool.

Study, year	Selection bias	Study design	Confounders	Blinding	Data collection method	Withdrawal and dropouts	Global rating
Brimelow et al, 2020 [24]	Weak	Weak	Weak	Moderate	Strong	Weak	Weak
D’Cunha et al, 2020 [26]	Weak	Strong	Weak	Moderate	Strong	Strong	Weak
Kang et al, 2021 [27]	Weak	Strong	Weak	Moderate	Strong	Strong	Weak
Moyle et al, 2018 [28]	Weak	Weak	Weak	Moderate	Strong	Weak	Weak
Brimelow et al, 2021 [25]	Weak	Weak	Weak	Moderate	Strong	Moderate	Weak
Saredakis et al, 2021 [29]	Strong	Moderate	Weak	Strong	Strong	Strong	Moderate

## Discussion

### Principal Findings

To the best of our knowledge, this is the first systematic review to evaluate the effectiveness, contents, and implementation of VR interventions for managing apathy in people living with cognitive impairment. A wide range of strengths and weaknesses were highlighted in the included studies (n=6). Our findings showed preliminary evidence that VR interventions can have a positive impact on apathy. However, the methodological limitations of the individual studies make it difficult to come to a firm conclusion. Even so, the findings showed that implementing a VR intervention may have a positive effect on apathy, regardless of the immersion level.

Among the 6 included studies, the largest effect size was found in 1 (17%) study using a large-screen display, which was categorized as a moderate level of immersion [28], followed by 2 (33%) studies using HMDs, which was categorized as a high level of immersion [24,27], whereas 2 (33%) studies used moderate-immersion VR. In the study by D’Cunha et al [26], a large projector screen was used instead of an HMD: the authors explained that using an HMD may isolate the user from the social environment, hindering the improvement in apathy. In the study by Moyle et al [28] too, a large-screen display was used for the VR intervention. Yet, the results are not consistent between the 2 studies, with one showing insignificant results [26] and another demonstrating significant improvement with a large effect size [28]. Therefore, we argue that content, rather than level of immersion, affects the intervention effects on apathy, supported by the evidence of the study by Saredakis et al [29], who compared the effects of the high-immersion VR group with the low-immersion laptop computer group that shared the same content and found that there was no significant difference between the groups. Our assumption may contradict the theories of García-Betances et al [13] and Witmer et al [14], who advocated that the higher the immersion level used, the greater the observed effect. More research in this area may be needed.

Natural scenes were widely used and preferred in the VR interventions. However, the studies (n=3) that solely used natural scenes showed conflicting results [24,26,28]. The studies (n=2) that showed significant improvement in apathy had played relaxing music during the VR session to enhance the participants’ pleasure [24,28], whereas the study that showed negative results required participants to engage in activities that involved physical strain without background music [26]. Therefore, we suspect that music combined with natural scenes may produce a better improvement in apathy. This is because pleasure derived from being immersed in a VR environment may increase participants’ engagement [36], whereas music has been proven to be a useful medium to improve the mood of people with cognitive impairment. In addition, the Attention Restoration Theory suggests that natural environments can restore mental fatigue by triggering spontaneous forms of attention; thus, being immersed in a natural environment can provide positive psychological effects, leading to a reduction in apathy among participants [37,38]. Even the authors of the study that reported insignificant results after exposing participants to natural scenes in VR have suggested integrating music into the design of the content of a future VR intervention [26]. Hence, it is assumed that including background music with a VR intervention may assist in reducing apathy by removing distractions from the real world during the VR session [28].

The included studies reported that participants appreciated and enjoyed the *realistic surroundings*, which were understood to be of high graphical fidelity and a colorful VR environment. Surprisingly, instead of providing a VR environment that was *as real as possible*, the study by Brimelow et al [24] adopted an approach that involved making intentional adjustments to the visual presentations, such as a high-contrast design (eg, a scene of penguins in the snow), to accommodate age-related visual decline [24]. By contrast, participants from another study complained that they had visuoperceptual difficulties recognizing the objects displayed on the screen and that the sound was too soft to be heard clearly [28]. Therefore, customized adjustments to legibility and auditory features in VR interventions created for people with cognitive impairment is advocated.

Of the 6 included studies, 2 (33%) investigated the effects of a game-based VR intervention on apathy. The study by D’Cunha et al [26] included a paddling task, and the study by Kang et al [27] used tasks involving multiple cognitive domains. Despite the high level of interaction, the study with the paddling task had relatively high dropout and incompletion rates [26]; 1 out of 10 participants withdrew before the intervention started, and 3 out of 10 participants from the intervention group stopped cycling shortly after commencing the VR intervention because of discomfort in the lower part of their body. It was presumed that the combination of VR and intensive motor training consumed more energy, making it difficult for the participants to continue, and could have resulted in a higher incompletion rate. By contrast, the intervention that used tasks involving multiple cognitive domains required minimal physical energy [27]; the increased interest and motivation of the participants was noted and might have contributed to higher engagement and a lower dropout rate. In future studies, interventions should be designed and implemented carefully whenever physical effort is required.

The facilitators of the VR interventions in the included studies were those who had prior knowledge of the participants, except for the study by Saredakis et al [29] in which the intervention was delivered by the researchers. The interventionist plays an important role in ensuring a good intervention outcome because inadequate trust in the interventionist would inhibit participants from engaging in the program [39]. Of note, a few adverse events were reported in the studies. This finding was similar to that reported in another systematic review of the use of VR for individuals with neurocognitive disorders [40]. This also implies that VR interventions are likely to be safe for people with cognitive impairment. Nevertheless, it is still important to note that some common forms of cybersickness such as eyestrain and blurred vision were reported, especially during a dynamic water scene [24]. Therefore, closely monitoring the side effects of VR on participants with cognitive impairment is needed because they may have limited ability to communicate their discomfort. Some measures such as avoiding sudden scene changes, replacing a dynamic scene with a static scene (eg, a farmyard), and asking participants to open their eyes slowly could be used to help them to adjust to a change in lighting and minimize symptoms of cybersickness [24,25,27,29].

It is worth noting that all included studies had a relatively small sample size, which pointed to the potential for selection bias and low statistical power. To provide solid evidence on the effectiveness of VR interventions, it is suggested that larger sample sizes should be adopted in further research. Moreover, of the 6 studies in this review, 5 (83%) were conducted in residential aged care facilities in Australia. Generalizing the results to more diverse settings or other nations could be the direction of future investigations. As 50% (3/6) of the studies merely conducted a single, short episode of the intervention, limited evidence was found to support the sustained effect of VR interventions. It is suggested that future studies should be designed to last longer and include multiple sessions. In addition, the participants had different levels of cognitive impairment, ranging from mild to severe. It is suggested that future studies should focus on a specific level of cognitive impairment to investigate the effect of VR interventions. Last but not the least, the ethnicity of the sample was not reported in all the studies; whether race would have an impact on the response is yet to be confirmed. As the studies were not of high quality, the findings on the significant impact of VR interventions should be interpreted with caution.

### Limitations

Although different databases and keywords were included in the search process, it is possible that articles written in languages other than English were excluded. In total, 4 relevant databases were searched; yet, some relevant articles might not have been identified. Although advice from a librarian was sought, in future research the search can be expanded to databases that include publications in other languages.

Another potential limitation was that only published studies were considered in this review. Existing VR applications have reached the stage of commercialization, which means that technology companies might not have published the trial results of VR applications for different target populations in the market [41]. It is possible that some relevant data may not have been captured because of their unpublished status.

### Conclusions

A total of 6 studies were included in the final analyses. This systematic review indicated that VR interventions are likely to be effective in reducing apathy and are unlikely to cause harm for people with cognitive impairment. Several recommendations on VR practices have been mentioned in terms of levels of immersion, social interactions, themes and the adjustment of content, physical effort, and the interventionist-resident relationship. As VR technology offers benefits for delivering safe, flexible, cost-effective, and repeatable interventions for patient care, it is believed that the scope of VR will be expanded with further technological innovations. However, the quality of the existing evidence is limited. To generate stronger scientific evidence on VR interventions, full power, large-scale, and high-quality studies need to be conducted.

## Appendix

Multimedia Appendix 1 Details of the search conducted in each database.

## Abbreviations

HMD: head-mounted display
PRISMA: Preferred Reporting Items for Systematic Reviews and Meta-Analyses
VR: virtual reality
