# Supplementary material for: Virtual Reality Intervention for Managing Apathy in People With Cognitive Impairment: Systematic Review
Source: JMIR Aging. 2022 May 11;5(2):e35224. doi: 10.2196/35224 (PMC9133981; doi:10.2196/35224)
Supplement: Multimedia Appendix 1 [file aging_v5i2e35224_app1.pdf]

Additional file 1. Search queries for each database.

Appendix I. Searching syntax on Embase

(dementia:ab,ti OR 'cognitive impairment':ab,ti OR 'alzheimer disease':ab,ti OR 'mild cognitive impairment':ab,ti) AND ('virtual reality':ab,ti OR 'head mounted':ab,ti OR simulation:ab,ti OR virtual:ab,ti) AND (apathy:ab,ti OR apathetic:ab,ti OR 'lack of initiati*':ab,ti OR 'lack of interest':ab,ti)

Appendix II. Searching syntax on CINAHL

(TI (Dementia OR Alzheimer disease OR mild cognitive impairment OR cognitive impairment) AND TI (virtual reality OR head mounted OR simulation OR virtual) AND TI (apathy OR lack of initiation OR lack of interest)) OR (AB (Dementia OR Alzheimer disease OR mild cognitive impairment OR cognitive impairment) AND AB (virtual reality OR head mounted OR simulation OR virtual) AND AB (apathy OR apathetic OR lack of initiati* OR lack of interest))

Appendix III. Searching syntax on PubMed

((Dementia [Title/Abstract]) OR (cognitive impairment [Title/Abstract]) OR (Alzheimer disease [Title/Abstract]) OR (mild cognitive impairment [Title/Abstract])) AND ((virtual reality [Title/Abstract]) OR (head mounted [Title/Abstract]) OR (simulation [Title/Abstract]) OR (virtual [Title/Abstract])) AND ((apathy [Title/Abstract]) OR (apathetic [Title/Abstract]) OR (lack of initiati*[Title/Abstract]) OR (lack of interest [Title/Abstract]))

Appendix IV. Searching syntax on PsycINFO

ab,ti([Dementia] OR [Cognitive impairment] OR [Alzheimer disease] OR [mild cognitive impairment]) AND ab,ti([virtual reality] OR [head mounted] OR [simulation] OR [virtual]) AND ab,ti([apathy] OR ab,ti[apathetic] OR [lack of initiati*] OR [lack of interest])
